# Virtual reality for rehabilitation and engagement in pediatric intensive care: a systematic review of feasibility, safety and outcomes

**DOI:** 10.3389/fped.2026.1934650

**Published:** 2026-08-31

**Authors:** Insu Choi, Hwa Jin Cho, Kibum Kim, Sunyong Yoo, Min-Keun Song

**Affiliations:** 1 Department of Pediatrics, Chonnam National University Medical School, Gwangju, Republic of Korea; 2 Department of Pediatrics, Chonnam National University Children’s Hospital, Gwangju, Republic of Korea; 3 Department of ICT, Hanyang University, ERICA Campus, Ansan, Republic of Korea; 4 Department of Intelligent Electronics and Computer Engineering, Chonnam National University, Gwangju, Republic of Korea; 5 Department of Physical & Rehabilitation Medicine, Chonnam National University Medical School, Gwangju, Republic of Korea

**Keywords:** feasibility, patient engagement, pediatric intensive care unit, rehabilitation, virtual reality

## Abstract

Children admitted to pediatric intensive care units (PICUs) are at risk of acquired weakness, delirium, and long-term functional and neurocognitive morbidity. Although early rehabilitation is recommended to mitigate these adverse outcomes, its delivery remains challenging. Virtual reality (VR) has been proposed as a means of enhancing engagement in rehabilitation in this setting. This systematic review evaluated the feasibility, safety, and outcomes of VR delivered to children during routine pediatric intensive care to support rehabilitation, participation in rehabilitation, or emotional regulation. Three databases were searched through 8 August 2026, and the reference list of one relevant scoping review was screened; two reviewers independently screened all records (Cohen's *κ* = 0.85). Six studies involving 188 children were included: one randomized pilot trial, one prospective single-arm trial, two cross-sectional single-arm studies, and two case reports. Risk of bias was high, serious, or critical in every appraised study, and the certainty of evidence was very low across all outcome domains. The randomized trial found higher consciousness scores at 2 weeks in the VR group but no difference in social and personal functional ability at 3 months. In uncontrolled studies, most children were rated as highly engaged and reported enjoying VR, while parents reported a calming effect more frequently than the children themselves; a transient increase in one heart rate variability parameter occurred during VR but was not sustained afterward. Adverse events were reported in four of the six studies at rates of 3.1% to 20%, comprising dizziness, nausea, visual discomfort, and mild irritability, with one serious event adjudicated as unrelated to VR; the two studies that applied pre-specified criteria recorded no events and one unrelated event, respectively. VR appears feasible and safe for hemodynamically stable, alert children in the PICU and is consistently associated with high engagement. However, whether VR improves functional outcomes remains uncertain. Eligibility, rather than tolerability, may be the principal barrier to implementation, and the available evidence applies to only a minority of the PICU population.

**Systematic Review Registration:**

https://www.crd.york.ac.uk/PROSPERO/view/CRD42024607286, PROSPERO, CRD42024607286

## 1 Introduction

Children admitted to pediatric intensive care units (PICUs) are at risk of developing a range of complications that can result in physical, cognitive, and psychosocial impairments (1, 2). Clinicians have long sought to prevent or mitigate these complications both during hospitalization and after discharge (3).

Rehabilitation is recognized as an important strategy for preventing or mitigating complications associated with critical illness in children (4). Early rehabilitation in the PICU is essential, as it may facilitate earlier discharge while reducing adverse outcomes (4). Specifically, early mobilization may reduce the risk of complications such as deconditioning, pressure ulcers, ventilator-associated pneumonia, and neurocognitive decline by promoting physical activity, sensory stimulation, and emotional engagement. These benefits may contribute to faster recovery and earlier discharge from the PICU (4). Despite its importance, early rehabilitation in PICUs remains underutilized due to medical instability, limited staffing, and difficulties engaging young children in rehabilitation activities (5–7).

In recent years, virtual reality (VR) has emerged as an innovative and immersive tool for enhancing engagement and functional outcomes during rehabilitation across various populations (8–10). In adult critical care settings, VR interventions have demonstrated potential benefits in improving participation, reducing anxiety, and promoting early mobilization (8–10). Similarly, in pediatric populations, VR has been reported to increase motivation and participation in physical therapy by transforming conventional exercises into interactive and gamified experiences (11–15). For example, VR-based early mobilization using head-mounted displays has been described for a small number of adolescents in PICUs, with reported feasibility and engagement (14). Moreover, in pediatric cardiac intensive care units, VR has been reported to facilitate active participation in rehabilitation sessions and improve the overall therapeutic experience (15).

Accordingly, VR has been proposed as a means of making rehabilitation more engaging in an environment where sustaining motivation can be challenging (11–13, 15). Whether VR can also alleviate practical barriers to rehabilitation in the PICU, such as limited staffing or the physical restrictions imposed by invasive devices, remains unclear and represents an important question addressed by this review. A recent systematic review of VR applications in intensive care medicine highlighted its potential role in improving rehabilitation adherence and psychological outcomes among critically ill patients (8). However, despite growing interest and promising pilot studies, evidence regarding the specific application, feasibility, safety, and clinical outcomes of VR-based rehabilitation in PICU settings remains limited (16–19).

This systematic review aimed to synthesize the available evidence on VR delivered to children during routine pediatric intensive care to support rehabilitation, participation in rehabilitation, or emotional regulation, with a focus on feasibility, safety, and clinical outcomes. A previous scoping review has mapped the use of extended reality across pediatric intensive care, including both clinical and educational applications (18). The present review addressed a narrower and distinct question (Among interventions delivered to patients as part of rehabilitation or to support participation in rehabilitation, what evidence is available and how certain is that evidence?). Accordingly, we applied design-specific risk-of-bias assessment tools and the GRADE approach, grouped findings by outcome domain, and verified all extracted data against the primary reports.

## 2 Methods

### 2.1 Study protocol registration

This review was prospectively registered in the International Prospective Register of Systematic Reviews (PROSPERO; identifier: CRD42024607286). The protocol was developed in accordance with the Preferred Reporting Items for Systematic Review and Meta-Analysis Protocols (PRISMA-P) guidelines.

### 2.2 Eligibility criteria (PICO)

Population (P): Children (<18 years) admitted to a PICU.

Intervention (I): VR delivered during routine pediatric intensive care to support rehabilitation, participation in rehabilitation, or emotional regulation, using either immersive or non-immersive systems. VR delivered as a procedural analgesic or anxiolytic adjunct, i.e., administered during, immediately before, or immediately after a specific painful or invasive procedure, was excluded because such applications address acute procedural distress rather than the rehabilitative or participatory goals of critical care recovery and have been examined in a separate body of literature.

Comparison (C): Conventional rehabilitation or standard care, when available.

Outcomes (O): Functional recovery, physical activity during the intervention, rehabilitation participation and engagement, emotional well-being, physiological response, safety, and feasibility of delivery. Any validated or study-specific measure of these domains was eligible, and the measure used in each study is reported.

We included randomized controlled trials, non-randomized interventional studies, cohort studies, case series, and case reports. Conference abstracts, reviews, commentaries, editorials, protocols, trial registry records without published results, and non-English language articles were excluded. Case reports were made eligible, and non-immersive systems were made eligible following protocol registration. The registered criteria had specified case series comprising three or more patients and did not address the level of immersion. A third amendment excluded VR delivered as a procedural analgesic or anxiolytic adjunct. These amendments are reported under PRISMA item 24c, and the PROSPERO record has been updated accordingly.

### 2.3 Search strategy

A comprehensive literature search was conducted in the Cochrane Central Register of Controlled Trials, MEDLINE (via PubMed), and Embase from inception to 8 August 2026, with no date restrictions and with results restricted to English-language records in MEDLINE and Embase. In addition, we screened the reference list of one relevant scoping review of extended reality in pediatric intensive care that was identified by the search and excluded at full text screening because it was a review. Trial registries were not searched directly; however, the Embase interface indexes records from the WHO International Clinical Trials Registry Platform and ClinicalTrials.gov, and records from these sources retrieved by our search were screened and excluded when they represented ongoing studies without published results. No conference proceedings or other grey literature sources were searched. The full search strategies as executed, including the record count for each search block, are presented in the Supplementary Appendix S1.

### 2.4 Study selection and data extraction

Two reviewers independently screened all titles and abstracts against the eligibility criteria. Full-text articles were retrieved when eligibility could not be determined from the title and abstract. Agreement was almost perfect (Cohen's *κ* = 0.85; raw agreement = 98.5%), and the two disagreements were resolved through discussion. Two reviewers independently extracted data using a standardized template, and all extracted data were verified against the primary full-text reports. Extracted items included study design, country and setting, population and eligibility criteria, screening and uptake data, illness severity, sample size and age, VR modality, dose and delivery, staffing requirements, outcome measures and results, and the method used to ascertain adverse events.

### 2.5 Risk of bias assessment

Two reviewers (I.C. and M.-K.S.) independently assessed the risk of bias. The randomized trial was appraised using the Cochrane Risk of Bias 2.0 tool. The non-randomized studies were appraised with ROBINS-I, which evaluates bias relative to a hypothetical target trial across seven domains. The two case reports were not formally appraised because instruments designed for comparative studies contain domains that are undefined in the absence of a comparison group, and applying such instruments would yield judgments that primarily reflect study design rather than study conduct. Therefore, their limitations are described narratively, and their findings are considered hypothesis-generating. Disagreements were resolved through discussion.

### 2.6 Data synthesis

**Figure 1:**
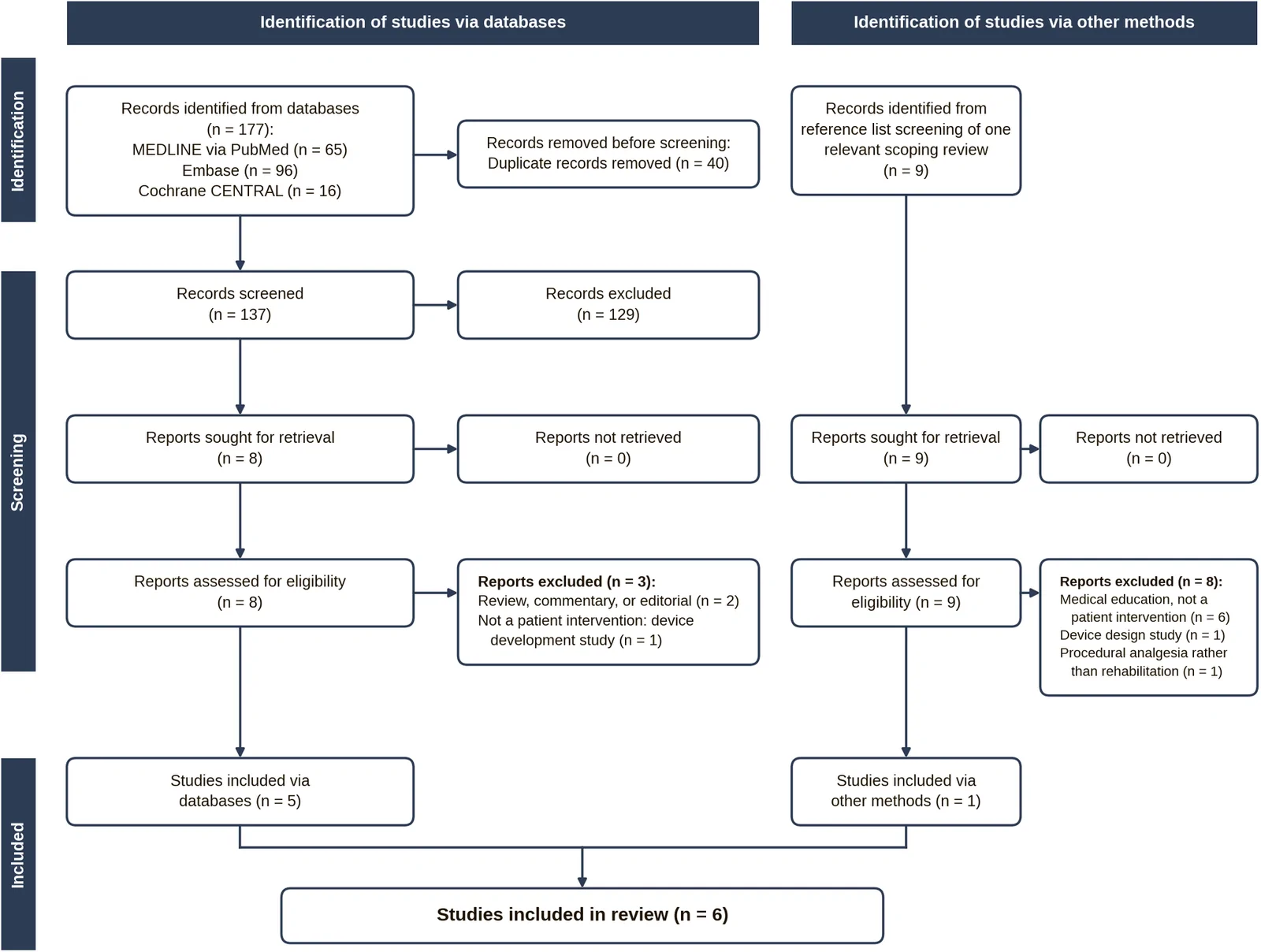
PRISMA 2020 flow diagram of study selection. Three databases were searched through 8 August 2026: Cochrane CENTRAL (Issue 7 of 12, July 2026), MEDLINE via PubMed, and Embase. The reference list of one relevant scoping review, which was itself identified by the search and excluded at full text screening, was also screened. Two reviewers independently screened all 137 records (Cohen's *κ* = 0.85; raw agreement = 98.5%). Reasons for exclusion at the title and abstract stage (*n* = 129) were as follows: not a VR intervention (*n* = 69); not a patient intervention, including medical education, staff training, or device development (*n* = 29); review, commentary, editorial, or protocol (*n* = 14); trial registry record without published results (*n* = 11); conference abstract or poster only (*n* = 2); not a pediatric intensive care population (*n* = 2); procedural analgesia or anxiolysis (*n* = 2). Trial registries were not searched directly; registry records indexed by Embase were screened and excluded.

**Figure 2:**
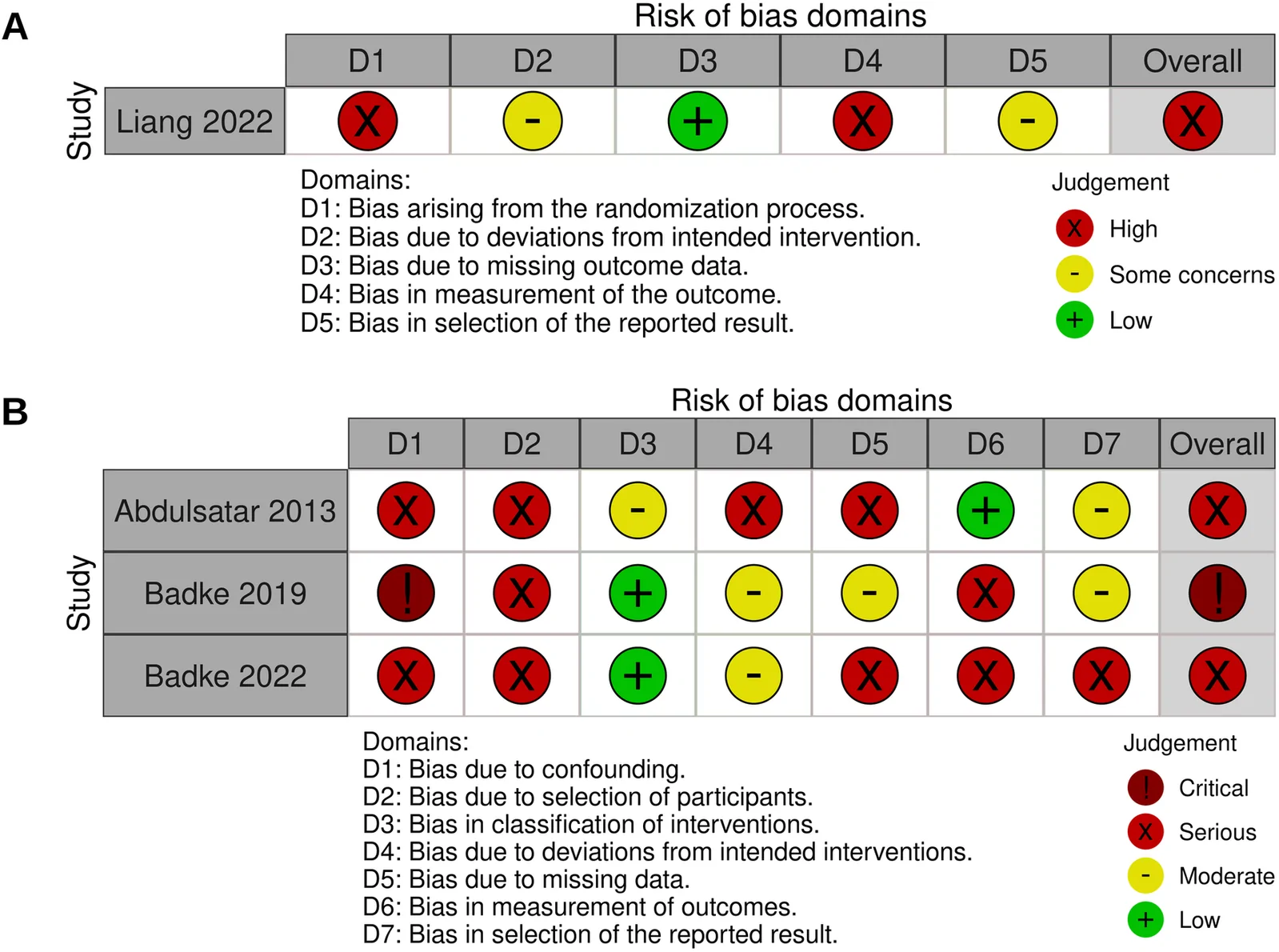
Risk-of-bias assessments of included studies. **(A)** The randomized trial was appraised using the Cochrane Risk of Bias 2.0 tool: D1, bias arising from the randomization process; D2, bias due to deviations from intended interventions; D3, bias due to missing outcome data; D4, bias in measurement of the outcome; D5, bias in selection of the reported result. **(B)** Non-randomized studies were appraised using ROBINS-I: D1, bias due to confounding; D2, bias in selection of participants; D3, bias in classification of interventions; D4, bias due to deviations from intended interventions; D5, bias due to missing data; D6, bias in measurement of outcomes; D7, bias in selection of the reported result. Judgments for RoB 2.0 were categorized as low risk, some concerns, or high risk; judgments for ROBINS-I were categorized as low, moderate, serious, or critical risk of bias. The two case reports were not formally appraised because instruments designed for comparative studies contain domains that are undefined in the absence of a comparison group; their limitations are described narratively in the Results.

Substantial clinical and methodological heterogeneity in study design, population, VR modality, dose, and outcome measures precluded quantitative synthesis. In addition, no two studies reported a common outcome measure at a common time point. Therefore, we conducted a structured narrative synthesis following the Synthesis Without Meta-analysis (SWiM) reporting guidelines. Studies were assigned to outcome domains according to the outcomes they reported: functional recovery, physical activity during the intervention, rehabilitation participation and engagement, emotional well-being, physiological response, safety, and feasibility of delivery. Within each domain we report the contributing studies, their designs and risk-of-bias ratings, and the direction and magnitude of effects as presented in the source reports. No summary effect measure was calculated, and no data conversion or imputation was performed. Where a source study did not report an estimable effect, this was recorded as not estimable. No formal subgroup analysis, meta-regression, or sensitivity analysis was performed because only six studies were available, and no subgroup hypothesis had been pre-specified at registration. Instead, studies were grouped descriptively according to immersion level and the nature of the activities required of the child. Study characteristics, findings according to domain, study selection, and risk-of-bias assessments are presented in Tables 1, 2, Figures 1, 2, respectively.

**Table 1:** Characteristics of the included studies.

Study (year)	Design	Country/setting	Population	n/age	VR intervention & session	Key findings
Liang et al. (16)	Randomized pilot trial	China/rehabilitation department, PICU, and SICU	Disorders of consciousness (vegetative or minimally conscious state) after brain injury	30 analyzed (32 enrolled)/2–16 y, median 6.1 y (VR) and 4.8 y (control)	Immersive, receptive. HTC Vive; personalized and panoramic 360° videos, 30 min/day for 2 weeks, added to general rehabilitation	CRS-R favored VR at 2 weeks (*p* = 0.003, remained significant after Bonferroni correction); GCS *p* = 0.045 (not retained after correctionn); aEEG not significant (*p* = 0.203); GOS-E Peds at 3 months not significant (*p* = 0.073). Adverse events: mild irritability (3/15)
Abdulsatar et al. (20)	Prospective single-arm pilot trial	Canada/pediatric critical care unit	Anticipated stay > 48 h; 40 of 140 screened children were eligible	12 enrolled, 8 received the intervention/3–16 y, median 11 y; median PRISM III score of 9.5; 4 of 8 mechanically ventilated	Non-immersive, active. Nintendo Wii Boxing; ≥10 min twice daily for 2 days; median total exposure of 54.5 min	Upper limb activity higher during play than the rest of the day (*p* = 0.049). Grip strength unchanged (*p* = 0.20). No change in vital signs. No adverse events according to pre-specified criteria. Only 2 of 8 participants completed the prescribed dose
Badke et al. (11)	Cross-sectional, single-arm pilot	USA/PICU, single center	Mixed PICU population; sedation, vasoactive support, encephalopathy, and clinical instability excluded (∼10% estimated to be eligible)	32 enrolled/3–17 y eligible, median 9 y	Immersive, receptive. Smartphone-based 360° video, single session capped at 15 min; median exposure of 6 min	All 31 respondents enjoyed VR; 84% wanted a longer duration. Calming reported by 82% of parents and 57% of children (36% unsure). Adverse events: dizziness (1/32)
Badke et al. (12)	Cross-sectional, single-arm, mixed methods study	USA/PICU, single center	Mixed PICU population; same exclusions as Badke et al. (2019); median PRISM III score of 3.0	115 enrolled, 95 with analyzable HRV/3–17 y, median 10 y	Immersive, receptive. Smartphone-based 360° video, single session; median exposure of 10 min	79% rated in the highest engagement category. Calming reported by 92% of parents and 78% of children. HRVi minimum higher during VR than before or after VR (both *p* < 0.001); pre vs. post-VR values did not differ (*p* = 0.387); HRVi median unchanged (*p* = 0.73). Adverse events: 10/115
Hemphill et al. (15)	Case report	USA/pediatric cardiovascular ICU	Post-heart transplantation, tracheostomy, continuous renal replacement therapy, hospitalized > 1 year	1/16 y	Immersive, active. HTC Vive with controllers; 3 sessions on hospital days 218, 281, and 288 (30, 20, and 10 min, respectively)	Engaged in physiotherapy after repeated refusal; achieved seated and dynamic standing activities; vital signs stable. No adverse events reported, no monitoring procedure described
Lai et al. (14)	Mini-ethnographic case study	USA/PICU	Adolescents deconditioned after critical illness; invasive ventilation excluded	2/15 y and 13 y	Immersive, active. Oculus Rift with adaptive software; 4 and 2 sessions (mean session duration of 18 and 35 min, respectively)	Both participants achieved moderate exercise intensity using arms only; improved affect and motivation to engage in mobilization. Each session required 2–3 staff, 17 min of setup, and 8 min of cleanup. Three adverse events were reported, including one serious event; all adjudicated as unrelated

Studies are ordered by design and then by publication year. Population entries describe the eligibility criteria reported by each study, and n/age indicates the number analyzed and the observed, rather than the eligible and age range. The VR intervention specifies the level of immersion and whether the intervention was receptive or active, as these characteristics determine the demands placed on the child. Key findings summarize results reported in the primary articles, including non-significant findings, together with adverse events and the methods used to ascertain them.

CRS-R, Coma Recovery Scale-Revised; GCS, Glasgow Coma Scale; GOS-E Peds, Glasgow Outcome Scale-Extended Pediatric Revised; HRVi, integer heart rate variability; ICU, intensive care unit; PICU, pediatric intensive care unit; PRISM, Pediatric Risk of Mortality; SICU, surgical intensive care unit; VR, virtual reality; aEEG, amplitude-integrated electroencephalogram.

**Table 2:** Summary of findings according to outcome domain.

Outcome domain	Studies (participants)	Findings	Certainty	Reasons for downgrading
Consciousness level (CRS-R, GCS)	1 randomized pilot trial (*n* = 30)	CRS-R favored VR at 2 weeks (*p* = 0.003, remained significant after Bonferroni correction); GCS, *p* = 0.045, did not remain significant after correction. Baseline consciousness distribution differed significantly between groups.	Very low	Risk of bias (baseline imbalance, no allocation concealment, unblinded subjective outcome assessment), imprecision, indirectness
Functional recovery	1 randomized pilot trial (*n* = 30); 1 single-arm trial (*n* = 8)	GOS-E Peds showed no between-group difference at 3 months (*p* = 0.073). Grip strength unchanged (*p* = 0.20); POPC worsened from baseline to discharge (*p* = 0.02).	Very low	Risk of bias, serious imprecision, no study powered for a functional endpoint
Physical activity during the intervention	1 single-arm trial (*n* = 8)	Accelerometer-measured upper limb activity greater during play than during the remainder of the day (*p* = 0.049; median 5-fold difference).	Very low	Uncontrolled within-person comparison, very small sample, single center
Rehabilitation participation and engagement	3 single-arm studies (*n* = 155); 2 case reports (*n* = 3)	79% rated in the highest engagement category; enjoyment 97%–100%. Case reports described participation after repeated refusal. No comparison condition; no validated engagement measure; session duration declined across successive sessions.	Very low	Study design, risk of bias, no active comparator (novelty effect not excluded)
Emotional well-being	2 single-arm studies (*n* = 147)	Calming reported by 82%–92% of parents and 57%–78% of children, with 36% unsure in the smaller study. Outcomes were assessed using unblinded investigator-developed instruments.	Very low	Study design, risk of bias, unvalidated subjective outcomes
Physiological response	1 single-arm study (*n* = 95); 1 single-arm trial (*n* = 8)	HRVi minimum higher during VR than before or after VR (both *p* < 0.001); pre vs. post-VR values did not differ (*p* = 0.387); HRVi median unchanged (*p* = 0.73); longer exposure associated with lower HRVi minimum. Vital signs unchanged after console-based play.	Very low	No control condition, surrogate outcome, inconsistent findings across parameters
Safety	6 studies (*n* = 188)	Two studies applied pre-specified criteria and recorded no events and one unrelated serious event. In the other studies, adverse event rates ranged from 3.1% to 20%, comprising dizziness, nausea, visual discomfort, and mild irritability. Two studies did not systematically assess virtual sickness.	Very low	Ascertainment not standardized, small samples, exclusion of the most severely ill children from all but one prospective study
Feasibility of delivery	1 single-arm trial (*n* = 8); 2 case reports (*n* = 3)	40 of 140 children screened were eligible; 2 of 8 children completed the prescribed dose, with intervention initiation at a median of 9.5 days after admission. Head-mounted sessions required 2–3 staff and about 25 min of setup and cleanup.	Very low	Descriptive data from small uncontrolled studies; no formal implementation outcomes

Certainty of evidence was assessed using the GRADE approach and is reported for each outcome domain together with the reasons for downgrading. Findings are presented as reported in the primary articles; no summary effect measure was calculated because outcomes were not pooled.

CRS-R, Coma Recovery Scale-Revised; GCS, Glasgow Coma Scale; GOS-E Peds, Glasgow Outcome Scale-Extended Pediatric Revised; HRVi, integer heart rate variability; POPC, Pediatric Overall Performance Category; VR, virtual reality.

### 2.7 Reporting bias

Tests for funnel plot asymmetry were not performed. Cochrane guidance advises against such tests when fewer than 10 studies are available because their statistical power is insufficient to reliably distinguish chance variation from genuine asymmetry. The possibility that studies with unfavorable findings remain unpublished is addressed narratively in the Discussion.

### 2.8 Certainty of the evidence

Certainty of the evidence was assessed for each outcome domain using the GRADE approach, considering risk of bias, inconsistency, indirectness, imprecision and publication bias. Ratings and the reasons for downgrading are presented in Table 2.

## 3 Results

### 3.1 Study selection

The searches identified 177 records: 65 from MEDLINE via PubMed, 96 from Embase, and 16 from the Cochrane Central Register of Controlled Trials. After removal of 40 duplicates, 137 records were independently screened by two reviewers, with almost perfect agreement (Cohen's *κ* = 0.85; raw agreement = 98.5%). Of these records, 129 records were excluded at title and abstract stage, most commonly because they did not describe a VR intervention (*n* = 69) or described medical education, staff training, or device development rather than a patient intervention (*n* = 29). Eight reports underwent full-text assessment, of which three were excluded, leaving five studies. Screening the reference list of one relevant scoping review (18), which had itself been identified by the search and excluded as a review, yielded nine additional records, of which one met the eligibility criteria. Therefore, six studies were included in the review (Figure 1). Four reports that might otherwise appear to meet the eligibility criteria were excluded for the following reasons: two studies delivered VR as an adjunct to a specific painful or invasive procedure rather than to rehabilitation or engagement; one study was conducted across a pediatric hospital in which intensive care accounted for only 16 of 213 VR encounters with no separable PICU subgroup (13); one report was a commentary without primary data.

### 3.2 Study characteristics

Table 1 summarizes the characteristics of the six included studies (11, 12, 14–16, 20). The locations of five studies were as follows: three in the United States, one in Canada, and one in China. One additional study from the United States was a two-case report. Study designs comprised one randomized pilot trial, one prospective single-arm trial, two cross-sectional single-arm studies, and two case reports. The study populations were heterogeneous and included children with disorders of consciousness after brain injury, general PICU admissions, a cardiovascular intensive care patient after heart transplantation, and adolescents with deconditioning following critical illness. Five studies used head-mounted displays, and one study used a console-based system. The interventions also differed in the demands they placed on the child: three studies delivered receptive video or sensory stimulation requiring no active cooperation, whereas three studies delivered active exergaming requiring alertness, cooperation, and upper limb function. Illness severity varied accordingly, with median PRISM III scores of 3.0 in the largest study and 9.5 in the study that included mechanically ventilated children.

### 3.3 Outcomes

#### 3.3.1 Functional recovery

Only the randomized trial (16) and the prospective single-arm trial (20) reported functional outcomes. In the randomized trial, social and personal functional ability measured using the Glasgow Outcome Scale–Extended Pediatric Revised (GOS-E Peds) did not differ between groups at 3 months (*p* = 0.073). Consciousness level assessed with the CRS-R favored the VR group at 2 weeks (*p* = 0.003), and this difference remained statistically significant after Bonferroni correction. In contrast, the between-group difference in the Glasgow Coma Scale (GCS) did not remain statistically significant after correction (*p* = 0.045), and amplitude-integrated electroencephalography did not differ between groups (*p* = 0.203). In the single-arm trial (20), grip strength did not change over the study period (*p* = 0.20), whereas the Pediatric Overall Performance Category worsened between baseline and discharge (*p* = 0.02), a finding more consistent with the clinical course of critical illness than with a demonstrable intervention effect. The certainty of evidence was very low.

#### 3.3.2 Physical activity during the intervention

One single-arm trial measured physical activity objectively (20). Upper limb activity measured by wrist accelerometry was significantly greater during Wii play than during the remainder of the same day (57.12 vs. 9.36 counts, *p* = 0.049; median 5-fold difference). This was the only objective measure of physical activity identified in the review, and the comparison was within-person and uncontrolled. The certainty of evidence was very low.

#### 3.3.3 Rehabilitation participation and engagement

Three single-arm studies (11, 12, 20) and two case reports (14, 15) contributed data on rehabilitation participation and engagement. In the larger cross-sectional study, 79% of children were rated in the highest engagement category on an investigator-developed scale with modest inter-rater agreement (77.3%), and 72% of children made positively valenced comments. The case reports described engagement in physiotherapy among children who had repeatedly declined participation, as well as participation for longer durations than during non-VR sessions. However, no study included a comparison condition, and no validated measure of engagement was used. Session duration declined across successive sessions in the only report that delivered more than one session. The certainty of evidence was very low.

#### 3.3.4 Emotional well-being

Two cross-sectional studies reported subjective calming during or after VR (11, 12). Parents reported that VR had a calming effect in 82% and 92% of cases, and children themselves reported being calmed in 57% and 78% of cases; in the smaller study, 36% of children were unsure whether VR had a calming effect. All instruments were investigator-developed and administered without blinding by the research team immediately after the session. The certainty of evidence was very low.

#### 3.3.5 Physiological response

One study measured integer heart rate variability (HRVi) before, during, and after VR in 95 children (12). The HRVi minimum was higher during VR than before or after VR (both *p* < 0.001); however, pre- and post-VR values did not differ (*p* = 0.387), indicating a transient change that did not persist after the intervention. The HRVi median did not differ across time points (*p* = 0.73). Longer VR exposure was associated with a lower, rather than a higher, HRVi minimum (*r* = −0.307, *p* = 0.002). In the single-arm trial (20), heart rate, respiratory rate, blood pressure, and oxygen saturation did not change after Wii play. The certainty of evidence was very low.

#### 3.3.6 Safety

Four of the six studies reported adverse events (11, 12, 14, 16); however, methods of adverse event ascertainment differed substantially, limiting direct comparison of event rates. Two studies applied pre-specified criteria. One study (20) used nine predefined physiological events, with relatedness adjudicated by the bedside nurse or physician, and recorded no events among eight children, four of whom were mechanically ventilated during the intervention. The other study (14) used an institutional definition and recorded three events for two children; one was classified as serious, and all were adjudicated as unrelated to the intervention. The remaining studies relied on investigator observation or individual questionnaire items and reported subjective symptoms in 3.1%, 8.7%, and 20% of participants, comprising dizziness, nausea, visual discomfort, and mild irritability. One study reported that no instrument for assessing virtual sickness was administered because the children could not communicate reliably, and one case report did not describe a monitoring procedure. No study evaluated VR during extracorporeal support or for hemodynamically unstable children. The certainty of evidence was very low.

#### 3.3.7 Feasibility of delivery

Eligibility appeared to be the principal constraint on implementation. In the only study reporting complete screening data (20), 40 of 140 children screened were eligible, 12 children provided consent, 8 children received the intervention, and 2 children completed the prescribed dose; the intervention was initiated at a median of 9.5 days after admission. The authors of a second study estimated that approximately 10% of PICU patients would have met their eligibility criteria (11). Resource requirements differed according to device type. The console-based system was set up once per session and subsequently delivered by caregivers, with an estimated device cost of approximately US$100. In contrast, head-mounted display sessions required two to three staff, approximately 17 min of setup and 8 min of cleanup, and nursing support for line and equipment management. No study directly measured staffing requirements or costs as outcomes. The certainty of evidence was very low.

The certainty of the evidence across all outcome domains is summarized in Table 2.

### 3.4 Risk of bias

The randomized trial (16) was judged to be at high risk of bias (Figure 2). Consciousness level differed significantly between groups at baseline (vegetative state in 33.3% of participants in the VR arm vs. 86.7% of participants in the control arm, *p* = 0.008), and allocation concealment was not described; accordingly, the randomization process was judged to be at high risk of bias. The outcome measurement domain was also judged to be at high risk of bias because the CRS-R and GCS are subjective behavioral scales assessed by an unblinded clinician. Among the non-randomized studies, Abdulsatar et al. (20) and Badke et al. (12) were judged to be at serious risk of bias. Both studies contained internal comparisons that supports descriptive but not causal inferences. Badke et al. (11) was judged to be at critical risk of bias because it contained no comparison of any kind. The two case reports were not formally appraised because comparative risk-of-bias instruments were not applicable; they described one and two patients selected on the basis of their clinical circumstances, without a control condition, blinding, or pre-specified outcomes, and thus provided no protection against selection or reporting bias. Their findings may be treated as hypothesis-generating throughout.

## 4 Discussion

This systematic review examined VR delivered to children during routine pediatric intensive care to support rehabilitation, participation in rehabilitation, or emotional regulation. Six studies met the eligibility criteria (11, 12, 14–16, 20): one randomized pilot trial, one prospective single-arm trial, two cross-sectional single-arm studies, and two case reports, published between 2013 and 2022 and involving 188 children in total. The certainty of evidence was very low across all outcome domains. The most consistent findings concerned engagement, enjoyment, and feasibility in selected patients; the evidence on functional recovery was limited to a single randomized trial, which found no between-group difference at 3 months.

### 4.1 Who can receive VR in the PICU?

The evidence from this review supports a narrower claim than is often made. VR appears feasible and well tolerated for critically ill children who are hemodynamically stable, alert, and free of equipment obstructing the face; however, these characteristics describe only a minority of the PICU population at any given time. Eligibility, rather than tolerability, appeared to be the principal constraint. In the only study reporting complete screening data, 40 of 140 children screened were eligible, and only 2 of 8 treated children completed the prescribed dose. The authors of a second study estimated that approximately 10% of PICU patients would have met their eligibility criteria. Recurring exclusion criteria included moderate-to-heavy sedation, vasoactive support, clinical instability, equipment obstructing the face, and age younger than 3 years, reflecting the clinical condition of the patient rather than limitations inherent to the technology. This creates a potential timing problem for early rehabilitation. Delivering VR within the first days of admission was feasible primarily for children with low illness severity and shorter stays, in whom the need for prolonged rehabilitative support may be limited. Conversely, waiting until more severely ill children have stabilized resulted in a median delay of 9.5 days in one study, by which time discharge may be imminent. The three studies that delivered VR as an adjunct to physiotherapy or early mobilization (14, 15, 20) addressed this issue differently, all involving children with prolonged critical illness and established deconditioning, with interventions initiated between day 5 and day 288 of admission. Two approaches could potentially broaden applicability. The first is the use of receptive rather than active VR. Active exergaming required alertness, cooperation and upper limb function; nevertheless, one trial (16) delivered receptive multisensory VR to children in a vegetative or minimally conscious state without reporting serious adverse events. The second is establishing the safety of VR for children receiving sedation and invasive mechanical ventilation, populations that were excluded from every prospective study excluded and have not been systematically evaluated. The median ages in the prospective studies were 9, 10, and 11 years, and the case reports described adolescents; thus, the available evidence primarily applies to school-age children and adolescents rather than to the broader PICU population.

### 4.2 Safety and what the evidence does not show

Adverse event ascertainment differed substantially across studies, making the reported rates unsuitable for direct comparison. Two studies applied pre-specified physiological criteria with adjudication of relatedness and recorded no events and one serious event judged unrelated to the intervention, respectively. The remaining studies relied on investigator observation or individual questionnaire items and reported subjective symptoms in 3.1% to 20% of participants. In one study, the authors reported that no instrument for virtual sickness was administered because the children could not communicate reliably. Therefore, the available evidence suggests that VR delivered to selected, clinically stable children is unlikely to cause clinically important physiological compromise, while mild transient discomfort may occur but remains poorly characterized. Multisensory enrichment has been proposed as a potential mechanism through which VR might support recovery (8, 17). However, this hypothesis is extrapolated from other populations: no included study directly assessed cortical activation or neuroplastic changes, and amplitude-integrated electroencephalography did not differ between groups in the only trial that measured it. Accordingly, the present evidence does not establish a neurophysiological mechanism underlying any potential rehabilitative benefit of VR.

### 4.3 Engagement, novelty, and the absence of active comparators

Engagement was the most consistent signal identified in this review; however, it requires cautious interpretation. The available evidence cannot determine whether the observed engagement is attributable specifically to VR or instead reflects the introduction of any novel and absorbing activity. No study included an active comparator, such as tablet-based games or music therapy, and most interventions consisted of a single short session. Where repeated sessions were reported, engagement duration declined across sessions, from 30 to 20 to 10 min in one case report (15). In the only study that examined a physiological correlate of exposure (12), longer VR exposure was associated with a lower rather than a higher heart rate variability minimum. These findings suggest that novelty or the initial attractiveness of the intervention may contribute to the observed engagement. Trials comparing VR with active controls and evaluating outcomes across repeated exposures are thus needed before improvements in engagement can be attributed specifically to the VR modality.

### 4.4 Relationship to previous work

A previous scoping review mapped the use of extended reality across pediatric intensive care, covering both clinical and educational applications (18). The present review differs in several aspects. First, its scope was restricted to interventions delivered directly to patients and, among these, to rehabilitation and engagement rather than procedural analgesia. Second, design-specific risk-of-bias tools and the GRADE approach were applied rather than instruments focused primarily on reporting quality. Third, findings were synthesized by outcome domain, with the certainty of evidence explicitly assessed for each domain. Finally, all extracted data were verified against the primary reports. Evidence from adult intensive care is cited in this Discussion solely as contextual background and is identified as such throughout; it was not combined with the pediatric evidence because eligibility constraints, developmental considerations, and outcome measures differ materially between the two populations.

### 4.5 Limitations and future directions

Two types of limitations should be distinguished: limitations of the available evidence and limitations of the review process. The evidence base was limited in several respects. All six studies were single-center studies, only one included a comparison group, sample sizes ranged from 1 to 115 participants, no study followed children beyond 3 months, none evaluated cost-effectiveness, and none used an active comparator.

The review process also had limitations. Our search strategy combined a VR concept block with compound phrases denoting pediatric intensive care and thus did not retrieve one included study whose title and abstract contained none of these phrases; that study was identified through reference list screening. The use of unqualified abbreviations such as VR, AR, and XR generated substantial retrieval noise, as AR frequently referred to animal research or autosomal recessive inheritance, whereas VR frequently referred to video review. We restricted inclusion to English language reports, did not search trial registries directly or grey literature, and amended the eligibility criteria after registration. These factors may have contributed to the omission of relevant studies and should be considered when interpreting the completeness of the evidence base.

Future reviews should consider separating pediatric, critical care, and VR concepts into three independent search blocks. Priorities for future research include randomized trials with active comparators and repeated exposures; evaluation of receptive VR for children unable to actively cooperate; formal assessment of safety during sedation and invasive mechanical ventilation; and use of standardized instruments for assessing adverse events and engagement.

## 5 Conclusion

VR appears feasible and well tolerated for selected critically ill children and is consistently associated with high levels of engagement and enjoyment. However, the available evidence does not demonstrate an effect on functional outcomes, and the children currently able to receive VR represent only a minority of the PICU population. Its clearest current application may be among children with prolonged critical illness who are clinically stable but disengaged from rehabilitation. Broadening its applicability will depend less on developing new VR content than on establishing whether VR can be delivered safely to sedated or mechanically ventilated children and on adopting standardized outcome and adverse event measures that enable meaningful comparison across future studies.

## Data Availability

The original contributions presented in the study are included in the article/Supplementary Material, further inquiries can be directed to the corresponding author/s.
