# Supplementary material for: Virtual reality for rehabilitation and engagement in pediatric intensive care: a systematic review of feasibility, safety and outcomes
Source: Front Pediatr. 2026 Aug 31;14:1934650. doi: 10.3389/fped.2026.1934650 (PMC13572598; doi:10.3389/fped.2026.1934650)
Supplement: Supplementary file 1 [file Supplementaryfile1.docx]

**Supplementary Appendix**

**Virtual reality for rehabilitation and engagement in pediatric intensive care: a systematic review of feasibility, safety and outcomes**

## 1. Databases searched and records retrieved

Three databases were searched on a single date, from inception, with no date limit. Results were restricted to English-language records in MEDLINE and Embase; the Cochrane Central Register of Controlled Trials does not apply a language limit in this search structure. In addition, the reference list of one relevant scoping review of extended reality in pediatric intensive care, itself retrieved by the search and excluded at full text as a review, was screened.

| **Database** | **Platform / interface** | **Date searched** | **Records** | **Language limit** |
| --- | --- | --- | --- | --- |
| MEDLINE | NLM / PubMed | 8 August 2026 | 65 | English |
| Embase | Elsevier | 8 August 2026 | 96 | English |
| Cochrane CENTRAL | Wiley (Issue 7 of 12, July 2026) | 8 August 2026 | 16 | None |
| Total before de-duplication |  |  | 177 |  |
| Duplicates removed | EndNote, Find Duplicates, with manual verification |  | 40 |  |
| Records screened |  |  | 137 |  |

Trial registries were not searched directly. The Embase interface indexes records from the WHO International Clinical Trials Registry Platform and ClinicalTrials.gov, and eleven such records were retrieved by our search, screened, and excluded as ongoing studies without published results.

## 2. Full search strategies as executed

Each strategy was run as three separate lines in the search history of the respective interface, and the third line combined the first two. Record counts for each line are given in section 3.

**MEDLINE (NLM / PubMed)**

```
#1  ("virtual reality"[tiab] OR "augmented reality"[tiab] OR "extended reality"[tiab]
     OR "mixed reality"[tiab] OR "head-mounted display"[tiab] OR VR[tiab] OR AR[tiab]
     OR XR[tiab] OR "Virtual Reality"[Mesh] OR "Augmented Reality"[Mesh])

#2  ("pediatric intensive care"[tiab] OR "paediatric intensive care"[tiab] OR PICU[tiab]
     OR "pediatric critical care"[tiab] OR "critically ill children"[tiab]
     OR "Intensive Care Units, Pediatric"[Mesh])

#3  #1 AND #2 AND english[Filter]
```

**Embase (Elsevier)**

```
#1  'virtual reality'/exp OR 'virtual reality' OR 'augmented reality'/exp
     OR 'augmented reality' OR 'virtual reality':ti,ab OR 'augmented reality':ti,ab
     OR 'extended reality':ti,ab OR 'head mounted display':ti,ab
     OR vr:ti,ab OR ar:ti,ab OR xr:ti,ab

#2  'pediatric intensive care unit'/exp OR 'pediatric intensive care':ti,ab
     OR 'paediatric intensive care':ti,ab OR picu:ti,ab
     OR 'critically ill child':ti,ab OR 'pediatric critical care':ti,ab

#3  #1 AND #2 AND [english]/lim
```

**Cochrane Library — CENTRAL (Wiley), entered through the Search Manager**

```
#1  ("virtual reality" OR "augmented reality" OR "extended reality"
     OR "mixed reality" OR "head-mounted display" OR VR OR AR OR XR):ti,ab,kw

#2  ("pediatric intensive care" OR "paediatric intensive care" OR PICU
     OR "critically ill children" OR "pediatric critical care"):ti,ab,kw

#3  #1 AND #2
```

Two asymmetries between the strategies are noted for transparency. The term "mixed reality" was included in the MEDLINE and Cochrane strategies but not in Embase. Controlled vocabulary was used where the interface supports it (MeSH in MEDLINE, Emtree in Embase); the Cochrane Library interface does not support MeSH restriction within this search structure, so keyword fields were searched instead.

## 3. Record counts for each concept block

These counts allow the search to be reproduced line by line and confirm that the combination in line #3 was applied.

| **Line** | **Concept block** | **Records** | **Database** |
| --- | --- | --- | --- |
| #1 | Virtual reality concept | 124,087 | MEDLINE |
| #2 | Pediatric intensive care concept | 46,146 | MEDLINE |
| #3 | #1 AND #2, English | 65 | MEDLINE |
| #1 | Virtual reality concept | 185,139 | Embase |
| #2 | Pediatric intensive care concept | 42,587 | Embase |
| #3 | #1 AND #2, English | 96 | Embase |
| #1 | Virtual reality concept | 20,402 | Cochrane CENTRAL |
| #2 | Pediatric intensive care concept | 2,480 | Cochrane CENTRAL |
| #3 | #1 AND #2 | 16 | Cochrane CENTRAL |

## 4. Screening and reasons for exclusion

After removal of duplicates, 137 records were screened independently by two reviewers. Agreement was almost perfect (Cohen’s κ = 0.85; raw agreement 98.5%, 135 of 137 records). The two disagreements concerned whether two commentary or review articles required full-text confirmation and were resolved by discussion.

| **Reason for exclusion at title and abstract** | **Records** | **Stage** |
| --- | --- | --- |
| Not a virtual reality intervention | 69 | Title/abstract |
| Not a patient intervention (medical education, staff training, device development) | 29 | Title/abstract |
| Review, commentary, editorial, or protocol | 14 | Title/abstract |
| Trial registry record without published results | 11 | Title/abstract |
| Conference abstract or poster only | 2 | Title/abstract |
| Not a pediatric intensive care population | 2 | Title/abstract |
| Procedural analgesia or anxiolysis rather than rehabilitation or engagement | 2 | Title/abstract |
| Total excluded at title and abstract | 129 |  |

Eight reports were sought and retrieved for full-text assessment from the database search, and nine from reference list screening. Reports excluded at full text are listed individually below, in accordance with PRISMA item 16b.

| **Report** | **Route of identification** | **Reason for exclusion** |
| --- | --- | --- |
| Caruso et al. (2020), Pediatr Qual Saf | Database search | Not a pediatric intensive care population. Conducted across a pediatric hospital; intensive care accounted for 16 of 213 VR encounters (7.5%) with no separable PICU subgroup, and 60% of use was perioperative. Eligible age range 6–18 years. |
| Kucher et al. (2020), Complement Ther Med | Database search | Procedural rather than rehabilitative. Nature-based VR delivered in the immediate postoperative period after total pancreatectomy with islet auto-transplant, targeting pain, nausea and anxiety. |
| Salem and Elokda (2014), J Pediatr Rehabil Med | Database search | Publication type. Labelled "Commentary" by the journal and subtitled as a commentary on the Abdulsatar pilot trial; presents no primary data. |
| Hoffman et al. (2019), Front Hum Neurosci | Reference list screening | Procedural rather than rehabilitative. VR delivered during burn wound debridement in the ICU hydrotank as an analgesic adjunct; outcomes were pain intensity and unpleasantness. |
| Natomi et al. (2021) | Reference list screening | Not a patient intervention. Design and validation of a device reproducing the sensation of holding an infant. |
| Raya et al. (2023), IEEE Comput Graph Appl | Database search | Not a patient intervention. Development and description of a VR application for delirium in the pediatric ICU; no sample size, outcome measure or statistical result is reported. |
| Ralston 2021; Yang and Oh 2022; Yu and Mann 2021; Dias 2021; Farra 2016; Farra 2019 (cost analysis) | Reference list screening | Not a patient intervention. Medical education or simulation training studies (6 reports). |

## 5. Included studies

Five studies were identified through the database search: Abdulsatar et al. (2013), Badke et al. (2019), Badke et al. (2022), Hemphill et al. (2021) and Lai et al. (2021).

One study was identified through reference list screening: Liang et al. (2022). This report contains no term denoting pediatric intensive care in its title or abstract; the setting appears only in the Methods, which is why the database search did not retrieve it. It appears as reference 29 of the scoping review whose reference list was screened.
